# The integration of multi-omics analysis and machine learning for the identification of prognostic assessment and immunotherapy efficacy through aging-associated genes in lung cancer

**DOI:** 10.18632/aging.205464

**Published:** 2024-01-22

**Authors:** Wei Lu, Yun Zhou, Ruixuan Zhao, Qiushi Liu, Wei Yang, Tianyi Zhu

**Affiliations:** 1 Department of Respiratory, General Hospital of Northern Theater Command, Shenyang, China; 2 Department of Respiratory, Wuhu Hospital, East China Normal University, The Second People’s Hospital of Wuhu, Wuhu, Anhui, China

**Keywords:** lung cancer, aging, WGCNA, prognostic model, immunotherapy

## Abstract

Background: Recent years revealed key molecules in lung cancer research, yet their exact roles in disease onset and progression remain uncertain. Lung cancer’s heterogeneity complicates prognosis prediction. This study integrates pivotal molecules to evaluate patient prognosis and immunotherapy efficacy.

Methods: The WGCNA algorithm identified module genes linked to immunity. The Lasso-Cox method built a prognostic model for outcome prediction. GO and KEGG analyses explored gene pathways. ssGSEA quantified immune cell types and functions. The riskScore predicts the effectiveness of immunotherapy based on its correlation with DNA repair and immune checkpoint genes. Single-cell sequencing examined key gene expression across cell types.

Results: Using WGCNA, we identified the MEbrown module related to immunity. Lasso-Cox selected “BLK,” “ITGB4,” “PRKCH,” and “SNAI1” for the prognostic model. MF analysis revealed enriched functions including antigen binding, GTPase regulator activity. In terms of BP, processes like immune signaling and mitotic division were enriched. CC enrichment included immunoglobulin complexes and chromosomal regions. Enriched pathways encompassed Cell cycle, Focal adhesion, Cellular senescence, and p53 signaling. ssGSEA evaluated immune cell abundance. RiskScore correlated with CTLA4 and PD1 through MMR and immune checkpoint analysis. Single-cell analysis indicated gene expression across cell types for BLK, ITGB4, PRKCH, and SNAI1.

Conclusion: In summary, our developed prognostic model utilizing age-related genes effectively predicts lung cancer prognosis and the efficacy of immune therapy.

## INTRODUCTION

Lung cancer is a malignant tumor that originates within lung tissue and ranks among the foremost causes of mortality worldwide [1, 2]. Therapeutic modalities for lung cancer encompass surgical intervention, radiotherapy, chemotherapy, targeted therapy, and immunotherapy, among others [3]. The realm of lung cancer treatment has experienced substantial advancements, particularly in the domains of molecular targeted therapy and immunotherapeutic interventions [4]. Molecular targeted therapy hinges on aberrant signaling pathways or specific mutations inherent to tumor cells, while immunotherapy involves activating the patient’s own immune system to combat malignant cells [5, 6]. Given the pronounced heterogeneity of lung cancer tissue, treatment outcomes exhibit marked variations [7, 8]. Therefore, there is an urgent need to introduce novel biomarkers and predictive models to achieve personalized therapeutic strategies for individuals.

Immunotherapy, as a revolutionary treatment approach, has garnered widespread interest and research in the fields of cancer and other diseases [9, 10]. In cancer immunotherapy, the primary strategies encompass checkpoint inhibitors, CAR-T cell therapy, cancer vaccines, and cytokine therapy [11]. Checkpoint inhibitors, a representative therapeutic method within immunotherapy, demonstrate remarkable efficacy in some patients [12]. Despite the notable successes achieved by immunotherapy, it remains challenged [13]. Some patients exhibit insensitivity to immunotherapy, while others experience immune system hyperactivity leading to adverse reactions such as autoimmune diseases [14, 15]. Hence, identifying patients sensitive to immunotherapy for targeted treatment is of paramount importance.

Senescence is a complex biological process that typically involves gradual degeneration and functional decline across various levels of an organism [16]. Many molecular mechanisms are believed to play pivotal roles in the senescence process [17]. Cellular dysfunction, oxidative stress, alterations in gene expression regulation, and shortened telomere length are factors profoundly influencing cellular functionality [18, 19]. Moreover, senescence has been found to play a crucial role in the initiation and progression of cancer [20]. Research indicates a close correlation between cellular senescence-related models and the prognosis of liver cancer patients [21]. Furthermore, neutrophil extracellular traps (NETs) formed by senescent neutrophils have been identified to facilitate breast cancer metastasis [22]. In the context of a Kras-driven lung cancer model, senescent macrophages assume a significant role in lung cancer genesis and progression [23]. However, current studies concerning the relationship between lung cancer and senescence often remain confined to singular molecular levels, with research exploring the mutual interplay between various key senescence-related genes and lung cancer being relatively scarce.

This study is founded on several crucial genes associated with senescence. It establishes a predictive model for lung cancer, employing a combination of bulk and single-cell sequencing analyses. The research delves into the potential correlations between this predictive model and immune cell infiltration. Furthermore, an evaluation of its implications in the realm of immunotherapy efficacy is conducted.

## MATERIALS AND METHODS

### Gene sets and data acquisition

Transcriptomic and clinical data for lung cancer were acquired from the TCGA database (https://portal.gdc.cancer.gov/), encompassing 526 cancer samples and 59 normal samples. The clinical dataset incorporates vital parameters such as survival status, survival time, age, gender, Stage, T, N, and M. Concomitantly, lung cancer dataset (GSE14814) was retrieved from the GEO database (https://www.ncbi.nlm.nih.gov/). After meticulous data curation, a total of 133 lung cancer samples were retained for subsequent model validation and analysis.

### WGCNA analysis

The Weighted Gene Co-expression Network Analysis (WGCNA) algorithm constructs co-expression networks by grouping genes with similar expression patterns into modules. This methodology adeptly unveils latent biological structures within high-dimensional gene expression data, transforming intricate data into interpretable modular information. This process thereby facilitates the identification of gene modules associated with specific biological processes or disease progression. In our study, we harnessed the power of WGCNA to construct a co-expression network using senescence-related genes. Subsequently, modules related to immunity were specifically selected for further in-depth analysis.

### Modeling analysis and validation

By employing Lasso-Cox regression analysis on the identified pivotal genes, we formulated a riskScore model, followed by the computation of individualized riskScore for the patients. This comprehensive evaluation gauges the role of these pivotal molecules in lung cancer patient prognosis. The specific formula for riskScore computation is as follows: riskScore = (Expression of BLK × coefficient) + (Expression of ITGB4 × coefficient) + (Expression of PRKCH × coefficient) + (Expression of SNAI1 × coefficient). The TCGA dataset was partitioned into training and validation sets. Furthermore, the GSE14814 dataset was employed for additional model validation. Kaplan–Meier (KM) survival curves were employed for survival analysis. These curves assess the existence of survival rate disparities between high- and low-risk groups of lung cancer patients within both training and testing sets. Risk survival curves were used to evaluate patient survival and mortality between high- and low-risk groups, as well as to assess differences in key genes of the constructed model between these two groups. ROC curves were employed to determine the predictive performance of the model univariable and multivariable Cox regression analyses were conducted to assess whether various clinical indicators serve as valuable independent prognostic factors.

### Functional enrichment analysis

Functional enrichment analysis using differentially expressed gene sets reveals key biological processes possibly implicated in distinct physiological or disease states. Gene Ontology (GO) and KEGG pathway analyses were conducted using the “clusterProfiler” R package. The GO analysis is categorized into three main aspects: Biological Process (BP), Cellular Component (CC), and Molecular Function (MF). The gene set files used for GO analysis annotation are derived from the c5.go.v7.4.symbols.gmt dataset. On the other hand, KEGG pathway analysis employs gene set files from the c2.cp.kegg.v7.4.symbols.gmt dataset (https://www.gsea-msigdb.org/).

### Analysis of the immune system and drug sensitivity

The ssGSEA algorithm quantifies the abundance of distinct immune cell infiltrations and immune functions within each sample. We evaluated a total of 16 immune cell types and 13 immune functions. The Estimate algorithm assesses the immune, stromal, and tumor components within the tumor microenvironment of each patient. Spearman correlation analysis was employed to calculate the correlation between the riskScore and MMR status, as well as immune checkpoint expression. This assessment helps determine the suitability of the riskScore for predicting the efficacy of immunotherapy. We evaluated the immunotherapy response concerning PD1 and CTLA4 in different riskScore groups. The “oncoPredict” package was utilized to evaluate the sensitivity of different riskScore groups to chemotherapy drugs. This evaluation encompassed commonly used chemotherapy drugs for lung cancer treatment.

### Mutation analysis

Single Nucleotide Variation (SNV) refers to the alteration of a single nucleotide within the genome, potentially leading to modifications in genetic information. Genetic mutation analysis contributes to the elucidation of gene functionality, regulatory mechanisms, and their associations with disease progression. The identification of key mutated genes within high-risk and low-risk groups aids in elucidating the prognosis of the disease.

### Analysis of single-cell data

The single-cell sequencing data originated from the GEO database (GSE149655). The “Seurat” package was employed to perform quality control, principal component analysis (PCA) dimensionality reduction, and t-SNE dimensionality reduction on the dataset, followed by clustering of the single-cell samples. The “SingleR” package was utilized to annotate cell types within the single-cell data, enabling the analysis of gene expression profiles across distinct cell populations. The “cellchat” package was employed to conduct cell communication analysis, investigating the molecular interactions between different cell types within the lung cancer tissue.

### Data statistics

The Wilcoxon test was employed for analyzing differences between two groups, while the Spearman correlation test was used for correlation analysis. Kaplan-Meier analysis along with log-rank test was utilized for survival analysis. Additionally, Cox regression analysis was performed using the R package “survival”, providing hazard ratios (HRs) and 95% confidence intervals (CIs). Statistical analyses and data visualization were conducted using R software (version 4.3.1).

### Data availability statement

The manuscript and accompanying supplementary material encapsulate the primary findings outlined within this investigation. For more information, kindly communicate with the designated corresponding author.

## RESULTS

### Integration of WGCNA and Lasso-Cox model construction

**Figure 1:**
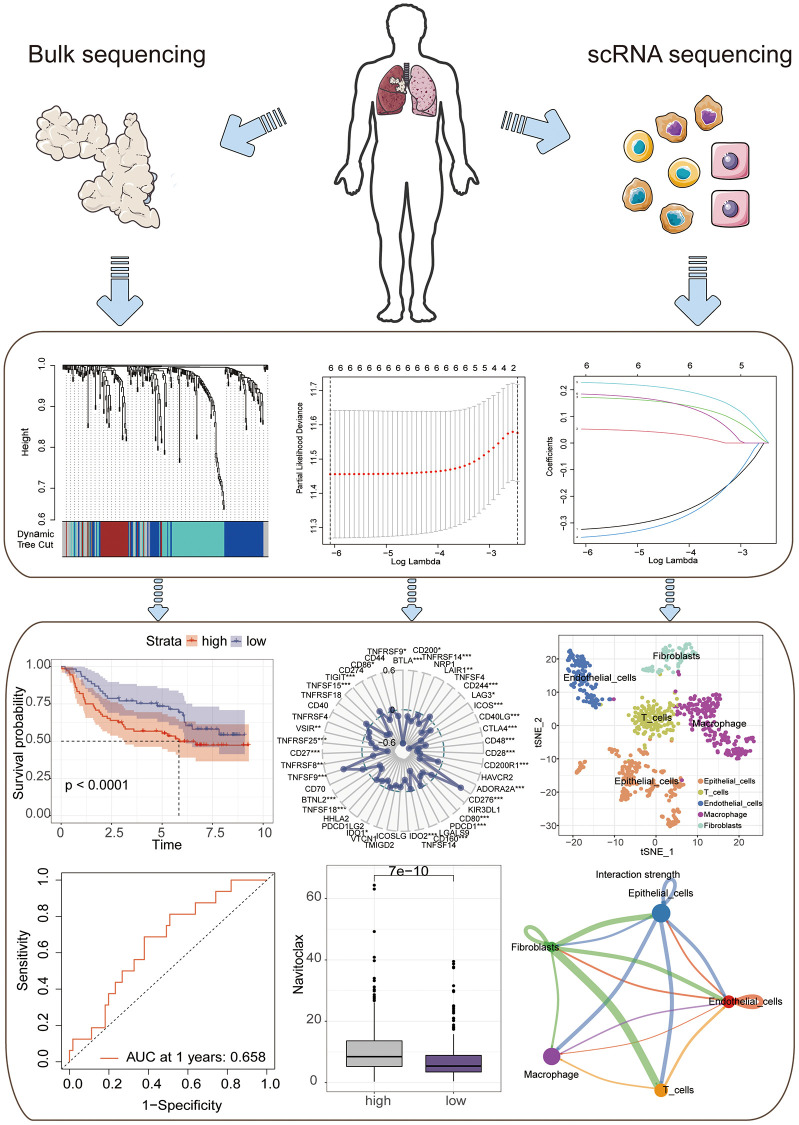
A flowchart of manuscript.

**Figure 2:**
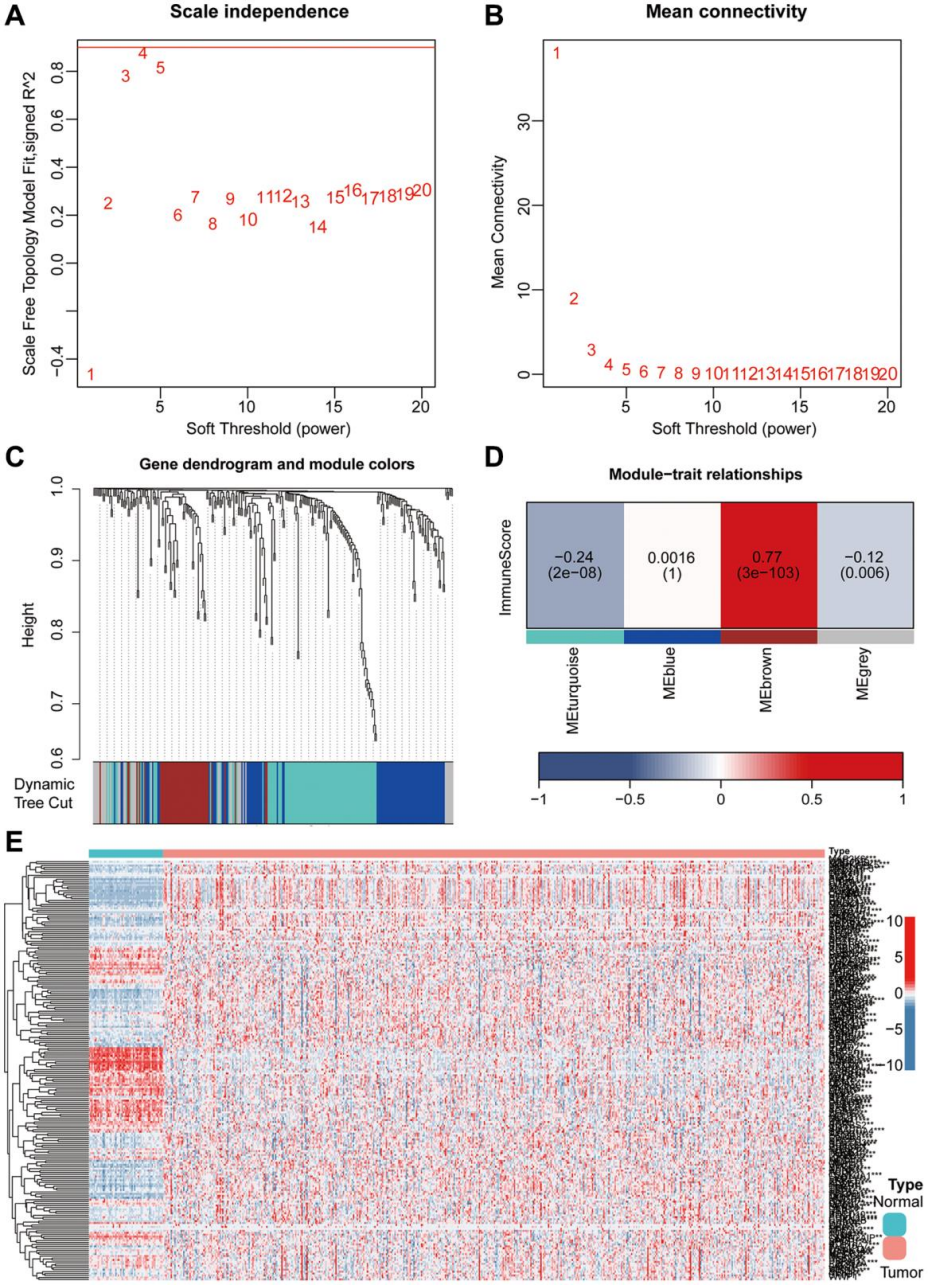
**He combined application of WGCNA and differential analysis was used to screen key genes associated with aging.** (**A**, **B**) The WGCNA algorithm demonstrated the optimal soft threshold. (**C**, **D**) The WGCNA algorithm identifies modules associated with immunity. (**E**) Heatmap showing differentially expressed aging-related genes.

**Figure 3:**
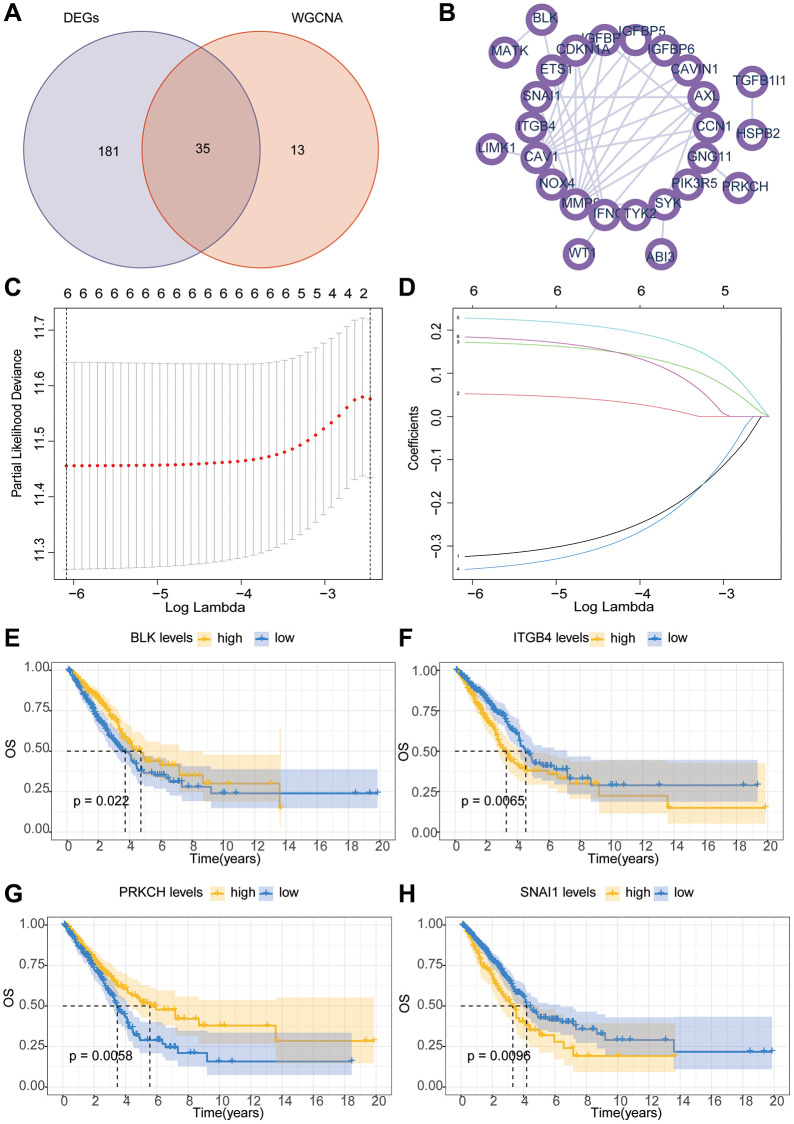
**The Lasso-Cox algorithm constructs a prognostic risk model.** (**A**) The Venn diagram illustrates the intersection of genes between WGCNA and differential analysis. (**B**) The PPI (Protein-Protein Interaction) network investigates the interactions between key proteins. (**C**, **D**) The Lasso-Cox algorithm constructs a model. (**E**–**H**) Kaplan-Meier survival curve analysis of hub gene.

Firstly, a flowchart was created to elucidate the comprehensive logical structure of the manuscript (Figure 1). In view of the potential correlation between senescence-associated genes and the pathogenesis and progression of lung cancer, we employed senescence-associated genes for the establishment of a risk prognosis model. To commence, we conducted clustering analysis on the senescence-associated genes using the Weighted Gene Co-expression Network Analysis (WGCNA) algorithm (Figure 2A, 2B). Notably, the genes were categorized into four distinct modules, with MEbrown displaying a close correlation with tumor microenvironment immune components. This specific module was subsequently chosen for further analysis (Figure 2C, 2D). Following this, we pursued the identification of genes manifesting significant distinctions between normal and tumor tissues (Figure 2E). By intersecting the genes from the MEbrown module, identified through the WGCNA algorithm, with the differentially expressed genes, a total of 35 genes were selected for subsequent modeling analysis (Figure 3A). Utilizing the STRING database, we further investigated the interplay among the genes within this intersection (Figure 3B). Subsequently, employing Lasso-Cox regression analysis, we discerned four pivotal genes, namely “BLK”, “ITGB4”, “PRKCH” and “SNAI1” for constructing the prognostic model (Figure 3C, 3D). Kaplan-Meier survival analysis distinctly demonstrated the intimate association of these key genes with the prognosis of lung cancer (Figure 3E–3H).

### Model validation

**Figure 4:**
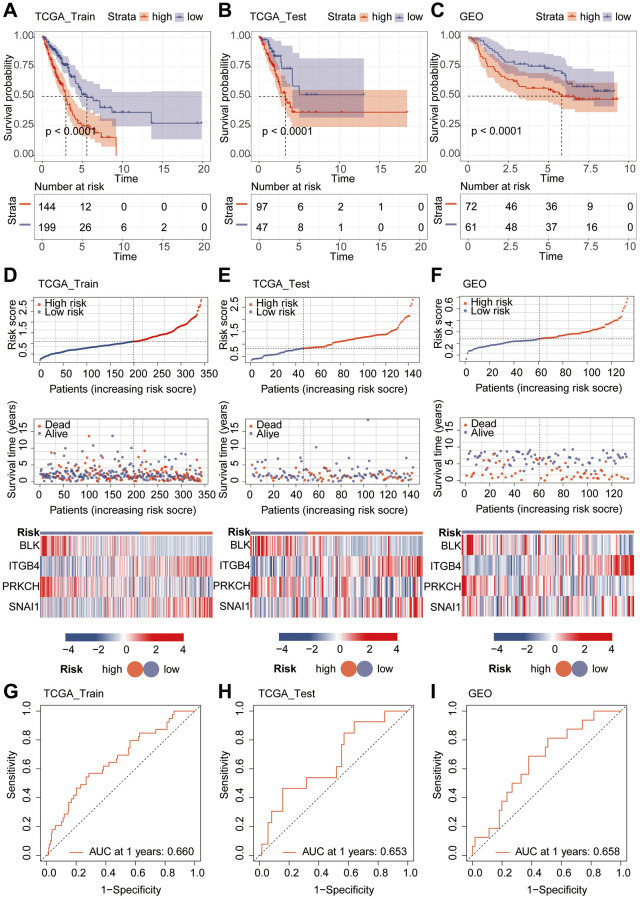
**Validation of model performance.** (**A**–**C**) Prognostic analysis using Kaplan-Meier curves for the riskScore model. (**D**–**F**) Heatmap and risk curve analysis of the riskScore model. (**G**–**I**) ROC analysis of the riskScore Model.

**Figure 5:**
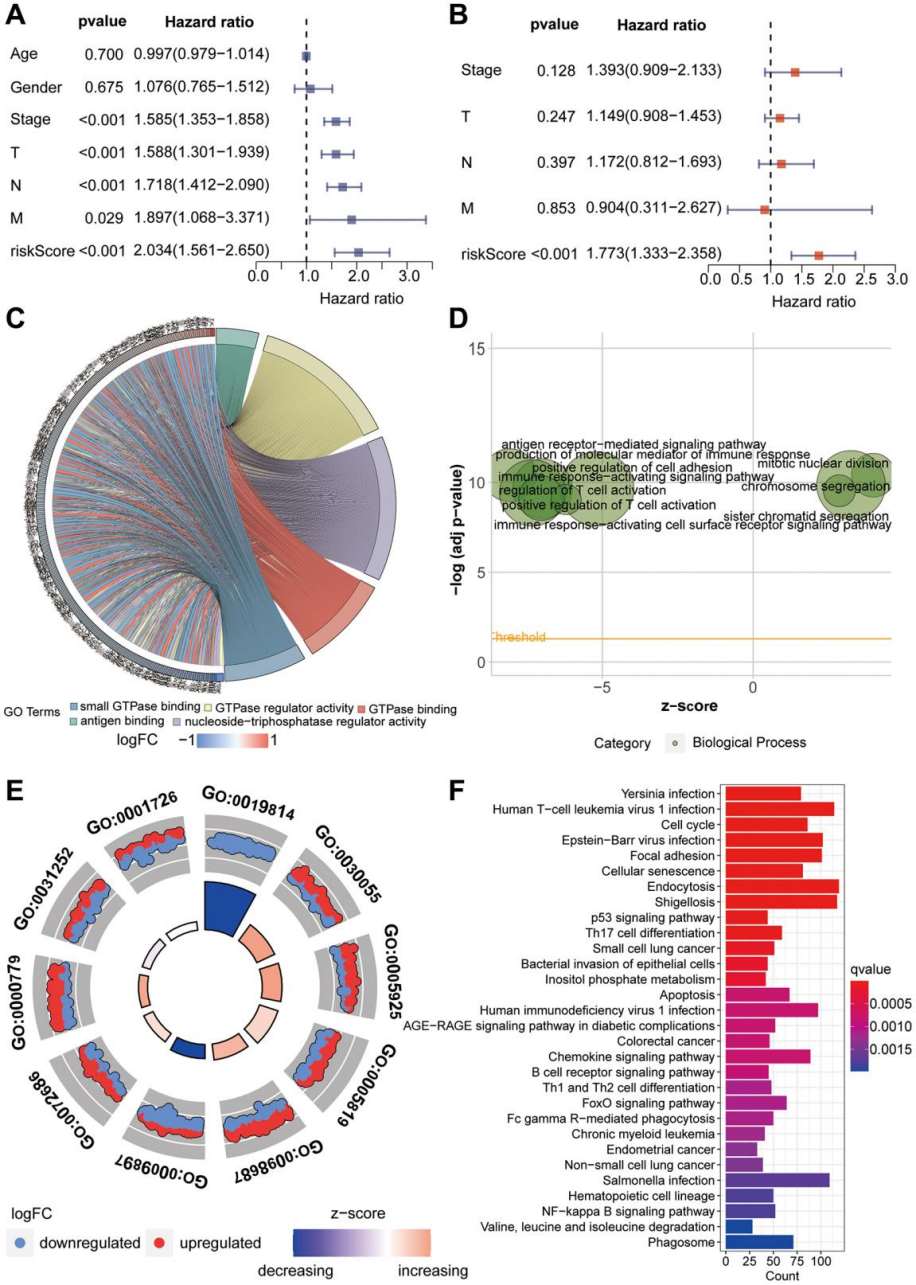
**Functional enrichment analysis.** (**A**, **B**) Univariate and multivariate Cox proportional hazards analyses of clinical indicators and riskScore. (**C**–**E**) Gene Ontology enrichment analysis of the riskScore model. (**F**) KEGG enrichment analysis of the riskScore model.

Given the interrelation between riskScore and patient prognosis, we undertook validation of the model’s prognostic predictive capability across three distinct datasets. The “caret” R package bifurcated the TCGA dataset into training and validation subsets, while the lung cancer dataset GSE14814 served as an additional validation subset. Notably, patients bearing high riskScore exhibited inferior prognoses within all three datasets (Figure 4A–4C). Further examination of survival status among varying risk groups exposed that individual in the high-risk category manifested higher mortality rates. Heatmap analysis unveiled a pronounced elevation in the expression levels of the genes “ITGB4” and “SNAI1” in the high-risk group, juxtaposed against lower expression levels of “BLK” and “PRKCH” in this group, when compared with the low-risk counterpart (Figure 4D–4F). The ROC curve, utilized to predict model classification performance, spotlighted the area under the ROC curve (AUC) as a metric for classifier efficacy. Notably, AUC values for TCGA training and validation subsets reached 0.660 and 0.653, respectively, while GSE14814 validation subset recorded an AUC of 0.658 (Figure 4G–4I). Subsequently, we proceeded with univariate and multivariate Cox regression analyses involving riskScore and clinical attributes. The univariate Cox analysis underscored the prognostic significance of Stage, T, N, M, and riskScore. Notably, the multivariate Cox regression analysis revealed that riskScore retained its pivotal role as an independent prognostic factor (Figure 5A, 5B).

### Functional enrichment analysis

GO enrichment analysis was employed to investigate the underlying reasons for significant differences between the high-risk and low-risk groups. In this analysis, the focus primarily rested on Molecular Function (MF), Biological Process (BP), and Cellular Component (CC). For Molecular Function, we observed significant enrichment in domains such as antigen binding, GTPase regulator activity, and nucleoside-triphosphatase regulator activity (Figure 5C). BP analysis unveiled enrichment in processes including the production of molecular mediators of immune response, antigen receptor-mediated signaling pathways, and mitotic nuclear division (Figure 5D). In terms of Cellular Component analysis, CC primarily enriched in areas such as immunoglobulin complex, cell-substrate junction and chromosomal region (Figure 5E). Furthermore, through KEGG enrichment analysis, we delved deeper into the potential mechanistic pathways contributing to the prognostic disparities between the high-risk and low-risk groups. Notably, pathways including Cell cycle, Focal adhesion, Cellular senescence, p53 signaling pathway and small cell lung cancer were prominently enriched (Figure 5F).

### Mutation analysis

**Figure 6:**
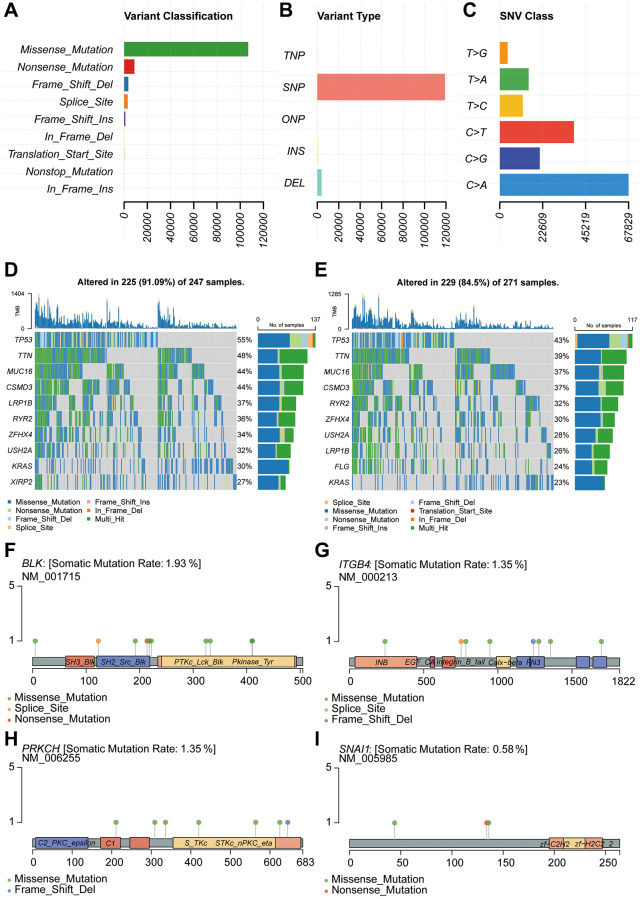
**Genetic mutation analysis for model construction.** (**A**–**C**) Mutation type analysis. (**D**, **E**) Analysis of mutated genes in high-risk and low-risk groups. (**F**–**I**) Analysis of single-gene mutation sites.

Genetic mutations refer to alterations in the composition or arrangement of base pairs in a gene's structure. We retrieved mutation-related data from the TCGA database for analysis. The analysis of variant classification revealed that the predominant category was Missense Mutation (Figure 6A). Furthermore, the analysis of variant types unveiled that the major category was Single Nucleotide Polymorphism (SNP) (Figure 6B). Regarding Single Nucleotide Variants (SNVs), the primary type identified was C>A (Figure 6C). Subsequently, aiming to delve into the mutation gene disparities between the high-risk and low-risk groups, we depicted a waterfall plot. The results indicated a substantial concordance in mutation genes between these risk groups (Figure 6D, 6E). For a more detailed examination of the mutation status of key genes within the constructed model, we performed somatic mutation rate analysis for each individual gene. The mutation rate for BLK was 1.93%, for ITGB4 it was 1.35%, for PRKCH it was 1.35%, and for SNAI1 it was 0.58% (Figure 6F–6I).

### Model immune landscape and drug sensitivity analysis

**Figure 7:**
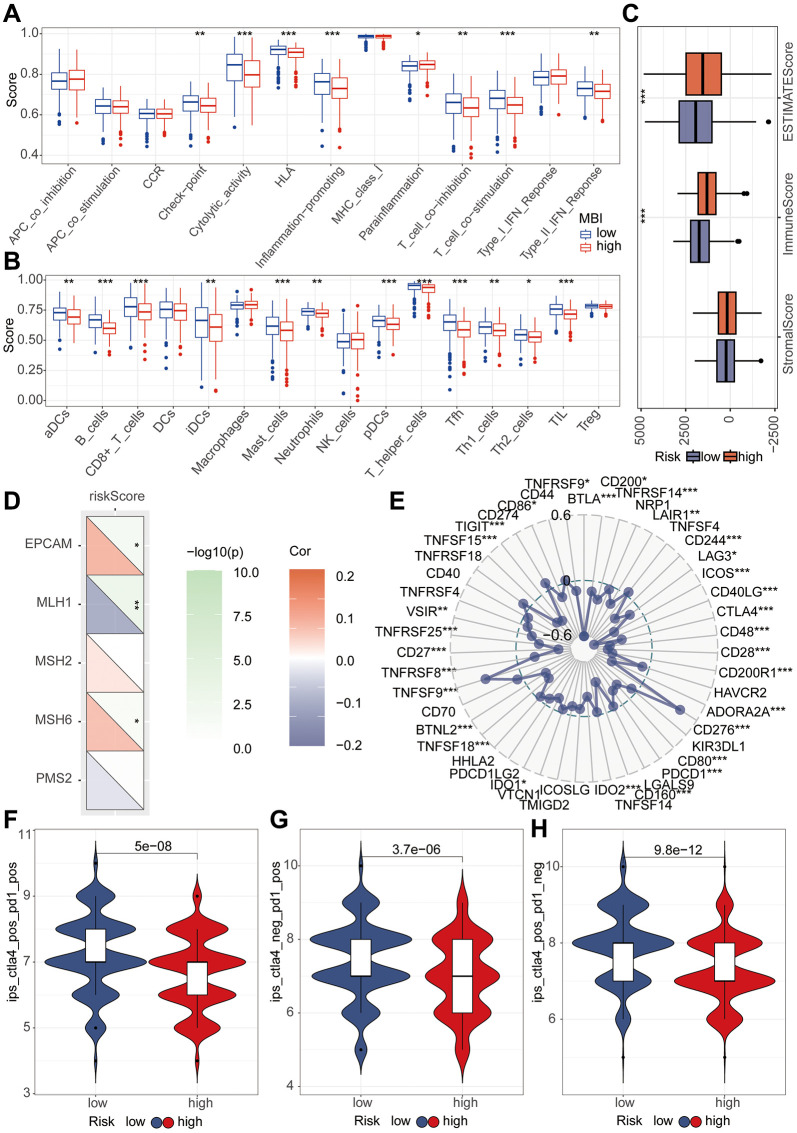
**Immune analysis of riskScore model.** (**A**) The ssGSEA algorithm was used to calculate the relationship between riskScore and various immune cells. (**B**) The ssGSEA algorithm was employed to assess the relationship between riskScore and different immune-related functions. (**C**) TME analysis was performed to evaluate the differences between high and low-risk groups. (**D**) Mismatch repair (MMR) analysis revealed a strong association between riskScore and MMR status. (**E**) Immune checkpoints analysis revealed a strong association between riskScore and immune checkpoints. (**F**–**H**) Immune therapy analysis reveals treatment efficacy in high and low-risk patient groups.

In pursuit of unraveling the relationship between riskScore and immune-related functionalities, we harnessed the ssGSEA algorithm to analyze 13 distinct immune-related functions. Evidently, Cytolytic activity, HLA, Inflammation-promoting, and T cell co-stimulation demonstrated significantly lower scores in the high-risk group compared to the low-risk group (*P* < 0.001) (Figure 7A). Subsequently, discerning the interplay between riskScore and immune cells, we delved into the abundance of 16 immune cell types within the tumor microenvironment. Notably, B cells, Mast cells, pDCs, T helper cells, Tfh, and TIL exhibited conspicuously reduced scores in the high-risk group as opposed to the low-risk group (*P* < 0.001) (Figure 7B). The tumor microenvironment (TME) encapsulates the milieu surrounding tumor cells, comprising vascular structures, immune cells, fibroblasts, bone marrow-derived inflammatory cells, diverse signaling molecules, and the extracellular matrix. The Estimate algorithm analysis unveiled a significantly elevated immune score in the high-risk group (Figure 7C). To comprehend the immunotherapeutic variance between high-risk and low-risk groups, we explored the potential correlation of this riskScore with MMR and immune checkpoint markers. The MMR analysis identified a positive correlation between EPCAM and PSH6 with the riskscore, while MLH1 exhibited a negative correlation (Figure 7D). Likewise, immune checkpoint analysis underscored the riskscore’s correlation with multiple indicators. Post-adjustment of p-values to 0.001, CTLA4 and PD1 continued to exhibit a close relationship with the riskscore, while PDL1 did not manifest any notable correlation (Figure 7E). For a more lucid observation of immune therapeutic conditions among high-risk and low-risk group patients, we conducted immune treatment score analysis. The results notably indicated higher immune treatment scores in the low-risk group (Figure 7F–7H).

**Figure 8:**
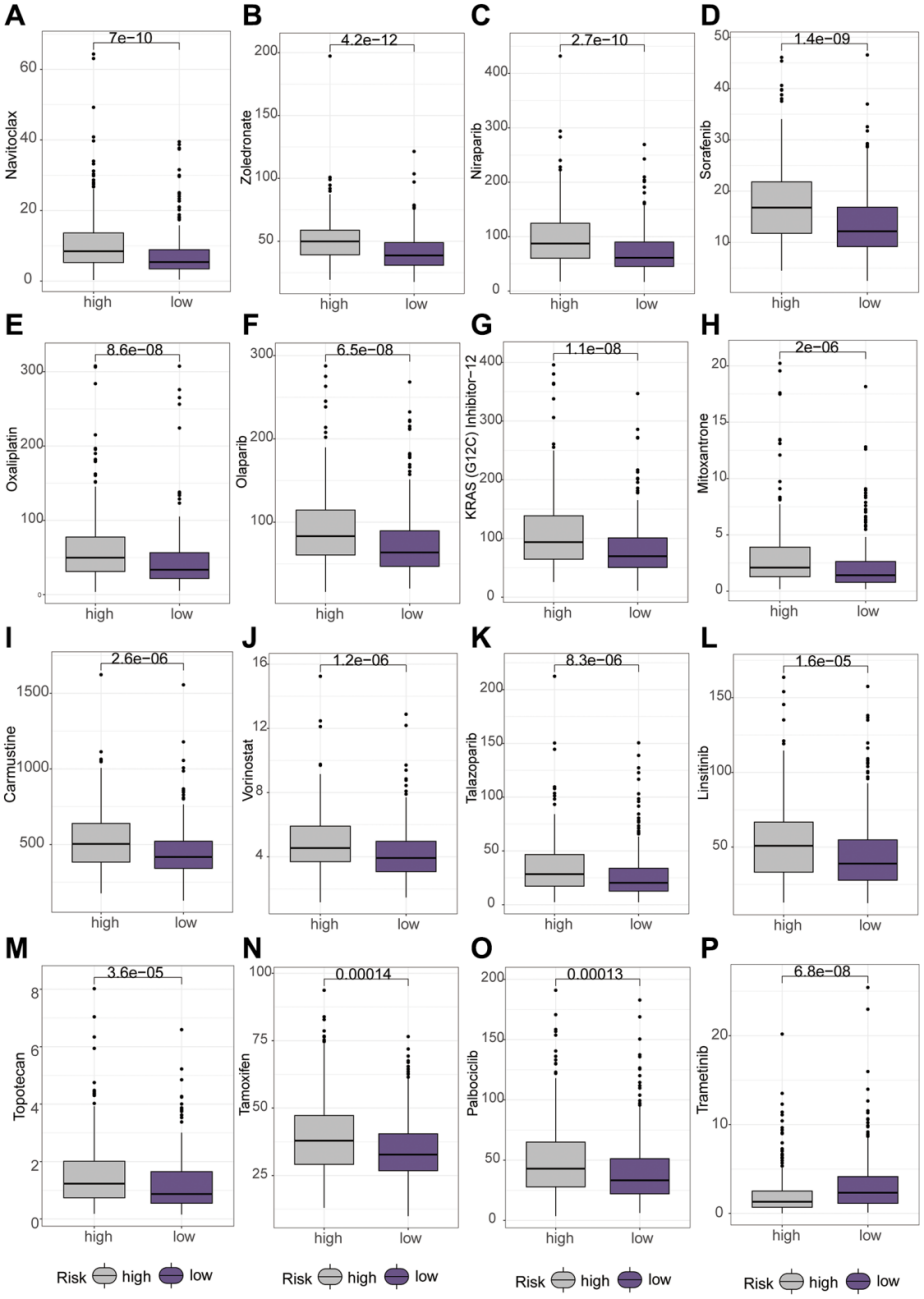
**Differential analysis of various chemotherapeutic drugs between high and low-risk groups using the “oncopredict” package.** (**A**–**O**) Higher IC50 values of different chemotherapeutic drugs in the low-risk group. (**P**) Higher IC50 values of chemotherapeutic drug in the high-risk group.

Subsequently, we delved into the sensitivity of high-risk and low-risk groups to diverse lung cancer chemotherapeutic agents. Interestingly, our findings revealed that Carmustine, KRAS (G12C) Inhibitor-12, Paclitaxel, Mitoxantrone, Navitoclax, Niraparib, Olaparib, Oxaliplatin, Palbociclib, Sorafenib, Talazoparib, Tamoxifen, Topotecan, Vorinostat, and Zoledronate exhibited higher IC50 values in the high-risk group (Figure 8A–8O). However, Trametinib displayed a higher IC50 value in the low-risk group (Figure 8P).

### Single cell data analysis

**Figure 9:**
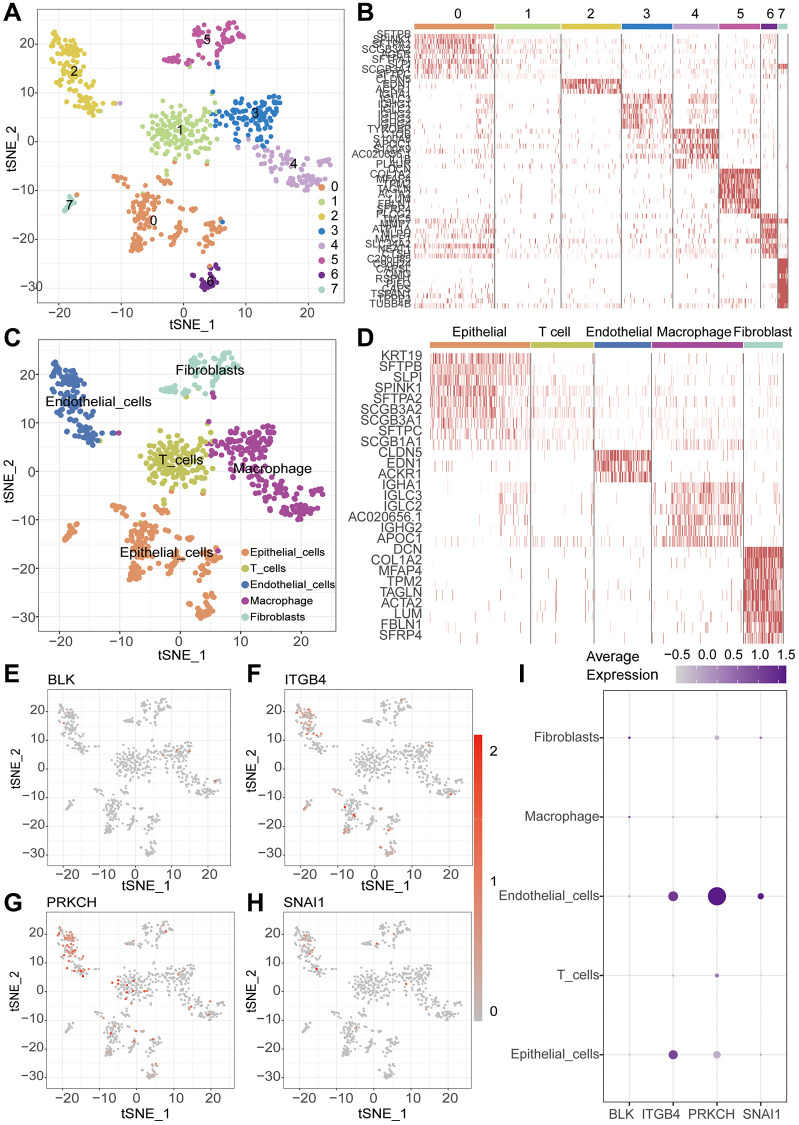
**Single-cell sequencing analysis.** (**A**) t-SNE clustering algorithm is used to classify cells into 8 clusters. (**B**) Heatmap visualizes the differentially expressed genes among the identified clusters. (**C**) “SingleR” package is employed to annotate different cell types, including T cells, Macrophage, Epithelial cells, Endothelial cells and Fibroblasts. (**D**) Heatmap visualizes the differentially expressed genes among the identified cells. (**E**–**I**) ScRNA-seq analysis reveals the expression patterns of key genes across different cell types.

**Figure 10:**
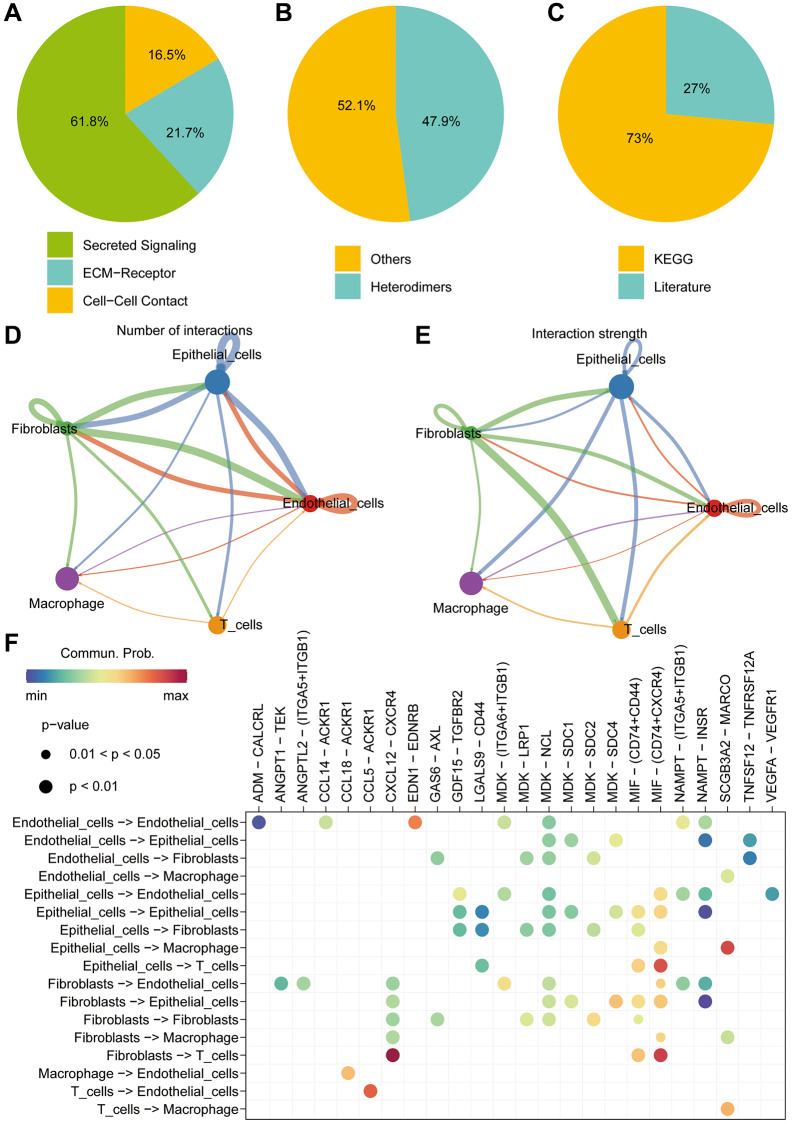
**Single-cell cell-cell communication analysis.** (**A**–**C**) Cell communication analysis reveals distinct communication patterns among different cell types in liver cancer. (**D**) The “cellchat” package is utilized to investigate the number of interactions between different cell types. (**E**) The “cellchat” package is employed to study the strength of interactions between different cell types. (**F**) The analysis focuses on exploring the molecular mechanisms underlying the interactions between different cell types.

With the continuous maturation of single-cell analysis techniques, their application in clinical diagnosis and treatment is expected to become more widespread. For example, the precise subtyping of tumors through single-cell sequencing technology is expected to provide a scientific basis for personalized treatment [24, 25]. We retrieved single-cell sequencing data of lung cancer patients from the GEO database (GSE149655). After data curation, a total of 2 lung cancer samples were included for subsequent analysis. Conducting PCA and tSNE dimensionality reduction analysis on the samples, we successfully stratified them into 8 clusters. Subsequent heatmap visualization highlighted the differential genes among these clusters (Figure 9A, 9B). Subsequently, cell annotation unveiled a clear categorization of cells into 5 main types: T cells, Epithelial cells, Macrophage, Fibroblasts, and Endothelial cells (Figure 9C). Additionally, we presented the differential genes among these diverse cell types, the heatmap allows us to distinctly observe the characteristic genes of different cell types (Figure 9D). Continuing our investigation, we analyzed the expression levels of key modeling genes across different cell types in lung cancer tissues. Through scatter plots, the expression of BLK was consistently low in all cell types. ITGB4 demonstrated predominant expression in Endothelia cells and Epithelia cells, while PRKCH exhibited primary expression in Endothelial cells. SNAI1, on the other hand, was notably expressed in Endothelial cells (Figure 9E–9I). Cellular communication is a crucial process of interaction among cells within an organism, involving the transmission of information between cells and the coordination of cellular behaviors. Cellular communication analysis, as a significant method for studying intercellular interactions, is of paramount importance in revealing the physiological and pathological processes of organisms [26, 27]. Further inquiry into cellular communication patterns revealed that Secreted Signaling accounted for 61.8%, ECM-Receptor for 21.7%, and Cell-Cell Contact for 16.5% of interactions (Figure 10A–10C). Among the interactions, those involving Smooth muscle cells and Monocytes, and the interactions between Fibroblasts and T cells, emerged as most prominent in terms of quantity and intensity (Figure 10D, 10E). Subsequently, we delved into the molecular interactions between distinct cell types. Notably, the interaction between Smooth muscle cells and B cells was mediated through the MIF signaling pathway, specifically involving CD74 and CXCR4 interactions. Similarly, the interaction between Smooth muscle cells and Monocytes also hinged on the MIF signaling pathway, facilitated by CD74 and CD44 interactions (Figure 10F).

## DISCUSSION

Lung cancer is a multifaceted disease, characterized by a multistep progression and involving the participation of numerous genes. It is influenced by a complex interplay of environmental and genetic factor [1, 28]. The presence of diverse subtypes and molecular subgroups within lung cancer adds to the complexity of its diagnosis and treatment, thereby presenting heightened challenges [2]. Immunotherapy has emerged as a revolutionary approach for cancer treatment, demonstrating notable efficacy in certain lung cancer patients. However, current research indicates that its effectiveness is confined to specific patient cohorts [14]. In the pursuit of refined and personalized interventions, the identification of biomarkers capable of predicting both the prognosis of lung cancer patients and the response to immunotherapy assumes paramount significance.

Due to the potential relevance of aging-associated genes in the pathogenesis of lung cancer, we employed the Weighted Gene Co-expression Network Analysis (WGCNA) algorithm to select aging-related genes for the construction of a Lasso-Cox model. The genes employed in the development of the prognostic model include BLK, ITGB4, PRKCH, and SNAI1. Notably, research findings substantiate the credibility of our gene selection. Specifically, BLK has been associated with inducing autophagy in bladder cancer cells, thereby impeding the progression of bladder cancer [29]. And Chen et al. have demonstrated that ITGB4, under the stimulation of KCNF1, promotes the proliferation of lung cancer cells and advances tumorigenesis [30]. The overexpression of PRKCH has been linked to the facilitation of neuroglioma occurrence and the modulation of neuroglioma stem cell characteristics [31]. Moreover, a body of research indicates that the upregulation of SNAIL initiates epithelial-mesenchymal transition, thereby facilitating tumor cell migration and lung cancer metastasis [32]. Subsequently, our riskScore model underwent comprehensive analysis utilizing three distinct datasets. The results consistently depict a notably inferior prognosis for patients in the high-risk group compared to their low-risk counterparts. Receiver Operating Characteristic (ROC) curves underscore the model’s robust predictive performance. Further, both single-factor and multi-factor Cox regression analyses underscore the value of this riskScore as an independent prognostic factor.

To further delve into the disparities in prognosis between the high-risk and low-risk groups, we employed GO analysis and KEGG analysis to unveil underlying biological processes and mechanistic pathways. In the context of GO analysis, notable enrichments were observed in nucleoside-triphosphatase regulator activity, mitotic nuclear division, and chromosomal region. It is noteworthy that mitosis is an indispensable process in cancer proliferation [33, 34]. Intriguingly, KEGG enrichment revealed pathways such as Cell cycle, Focal adhesion, p53 signaling pathway, and small cell lung cancer. Cancer, characterized by uncontrolled cell division, is intricately associated with disruptions in the cell cycle [35]. The significance of the p53/AMPK/mTOR pathway in the pathogenesis of lung cancer has been substantiated in research [36]. Remarkably, these findings align closely with the outcomes of our enrichment analyses. Additionally, mutation analysis revealed a remarkable convergence in mutated genes between the high-risk and low-risk groups.

Through the utilization of the ssGSEA algorithm, we extensively probed the relationship between riskScore and 16 types of immune cells, as well as 13 immune-related functionalities. Notably, Cytolytic activity, HLA, Inflammation-promoting, and T cell co-stimulation were found to exhibit significantly lower scores in the high-risk group compared to the low-risk group. Immunotherapy stands as a burgeoning approach in contemporary cancer treatment. Given the substantial heterogeneity among different tumor patients, identifying biomarkers capable of predicting immunotherapeutic efficacy remains of paramount significance [9, 11]. The utility of Mismatch Repair (MMR) as a common prognostic marker for immunotherapy outcomes is well established [37]. In our study, a remarkably strong correlation was observed between the riskScore and several genes, namely EPCAM, MLH1, MSH6, with MLH1 displaying the highest correlation. Notably, prior research has suggested a potential link between the gene polymorphism MLH1 −93A>G and the etiology of lung cancer [38]. Subsequently, a comprehensive assessment of the association between the riskScore and immune checkpoints was performed. The outcomes revealed a pronounced connection between CTLA4 and PD1 with the riskScore, while PDL1 failed to exhibit a significant correlation. This underscores the importance of considering PD1 and CTLA4 in clinical medication decisions. This observation was further substantiated by our extended analysis of immunotherapeutic trends.

Single-cell sequencing analysis is an advanced genomic technique enabling researchers to perform high-resolution exploration of individual cell gene expression, thereby revealing heterogeneity within cell populations [39]. In order to investigate the expression patterns of the pivotal genes in diverse cell types, we employed a single-cell analysis using the GEO dataset. Notably, ITGB4 exhibited elevated expression levels in both Endothelial cells and Epithelial cells, whereas PRKCH and SNAI1 demonstrated predominant expression within Endothelial cells. Cellular communication analysis, as a crucial method for studying intercellular interactions, is of significant importance in revealing the processes of life and the mechanisms of diseases. By comprehensively applying biochemical experimental methods and computational analysis approaches, we can dissect the regulatory networks of cellular communication at the molecular level, providing new insights and tools for biomedical research [26, 27]. Cellular communication plays a pivotal role in coordinating various physiological and biochemical processes among cells through signal transduction and interactions [40]. We meticulously examined cellular communication and potential molecular interactions among distinct cell types within lung cancer tissue. We found that cancer cells play a crucial role in the process of cell communication, and they interact with T cells, Macrophage, Fibroblasts, and Endothelial cells. Our results reveal the interacting molecules among different cell types. This analysis provides valuable insights directing our subsequent mechanistic exploration.

Certainly, it is imperative to acknowledge several limitations inherent in our study. To begin with, the reliability of bioinformatics heavily relies on the quality of data. The presence of subpar experimental data, sequencing errors, and other confounding factors can significantly impact the accuracy and dependability of the analyses. In light of this, it is imperative for us to conduct subsequent sequencing and analyses using our own samples to enhance data quality. Moreover, our study assumes a retrospective design. In the future, a prospective approach might be warranted to delve further into the explored aspects. Lastly, we did not delve into comprehensive mechanistic explorations downstream of the model genes. This aspect could potentially influence the precision of our predictions regarding immunotherapeutic efficacy and targeted medications. Therefore, an in-depth investigation is warranted to address these aspects. In an overarching context, this research holds substantial clinical applicability. We achieved this by combining bulk sequencing with single-cell sequencing in a comprehensive joint analysis. The riskScore formulated through the integration of WGCNA and Lasso-Cox methods emerges as a pivotal biomarker for prognosticating lung cancer patients’ outcomes. Importantly, this riskScore serves as an effective predictor for both immune therapy response and the efficacy of diverse chemotherapy agents. Collectively, these findings provide a substantial impetus toward advancing personalized immune therapy for lung cancer patients, thereby elevating the prospects of tailored treatment strategies.

## CONCLUSION

In conclusion, our endeavor has culminated in the development of a model centered around pivotal genes, capable of prognosticating lung cancer outcomes. Significantly, this model demonstrates adeptness in predicting the efficacy of immunotherapeutic interventions for patients.
